# Prevention in the Times of Crisis: A Look Back for the Way Forward

**DOI:** 10.1007/s10935-022-00677-0

**Published:** 2022-04-05

**Authors:** Samuel Tomczyk

**Affiliations:** Department of Health and Prevention, Institute of Psychology, University Greifswald, Greifswald, Germany

Recent years have brought multiple crises and global challenges to light: financial hardships, economic crises, climate change, the COVID-19 pandemic and, most recently, the war in Ukraine (to name a few). These crises are connected to biopsychosocial and societal burden, and their management requires resilient, collaborative and positive governance in a global community. The crisis management cycle identifies preparedness, response, recovery and mitigation as important stages of dealing with crises.

As prevention scientists, practitioners and policy-makers, we ask ourselves how we can contribute to this process. Naturally, prevention and health promotion are connected to the preparedness of populations as they strengthen resources and build resilience. Prevention also plays a role in response, recovery and mitigation, for example by supporting adaptive coping skills, providing psychoeducation and promoting health behaviors.

Moreover, these crises are an opportunity for learning and development in our discipline. As prevention professionals, we are aware of the enormous human suffering and the potential for additional environmental harm, but we also aim to create opportunities for positive and sustainable change. Reducing physical and often also social contacts during the COVID-19 pandemic, for instance, led to innovative digital solutions and digitalized behavior change interventions to support communication, health, and healthy relationships across physical distances. The field of prevention science was forced to think about new ways to interact, conduct research and implement and evaluate interventions.

As another example, the shared concern about climate change fosters new opportunities for multi-sectoral cooperation and interdisciplinary and global action, for instance, in international working groups on prevention, public and mental health, cross-continental research projects and calls to action. The most recent crisis, the war on Ukraine, reveals the potential of global digital communities and their impact on social support and the prevention of violence, crime and hate. Taken together, these challenging times tell us a lot about the human condition, from extraordinary strain to exceptional bravery and endurance.

Best practices and lessons learned from these events are also among key questions of the European Society for Prevention Research (EUSPR) in its journey with the Journal of Prevention. As a society for prevention scientists, practitioners and policy-makers across Europe, we want to explore, innovate, and improve the reach and impact of prevention from a cross-sectoral, cross-cultural and interdisciplinary perspective, for instance by preserving mental health, fostering positive and healthy youth development, and creating nurturing, sustainable and safe environments. With regards to current crises, the EUSPR asks:

- What have we, as prevention professionals, learned from these crises for our personal and professional lives, and what implications do we see for the field of prevention science?
- How has prevention science helped us to foresee, respond, recover, and mitigate, and how can we improve the contribution of prevention science not only on an individual but also a social, societal and even global level in the critically decisive years to come?
- How can research on the effective ingredients of interventions and policies be applied to rapidly emerging challenges and problems, i.e. how can we contribute to the resilience of our cultures, societies and families under immediate stress or even threat?
- Which directions is prevention science going in, and how are more traditional approaches and topics like individual health behavior change, and substance use, connected to global issues (e.g., climate change), policies and communities?

These are some of the questions that we would like to invite you to investigate, reflect on and discuss with us in the Journal of Prevention. Apart from these topical discussions, the Journal of Prevention also covers focused research on risk and protective factors, health and risk behaviors, determinants and etiology of positive and negative individual and social health outcomes as well as the development, implementation and evaluation of preventive interventions or local policies, the synthesis of evidence (like systematic reviews and meta-analyses), and debates on current and future directions in the field. Together, we want to examine the current state of the art of prevention science and practice, develop and discuss ways forward into an increasingly cyberphysical future, and create sustainable networks of scientists, practitioners and the public for the future or prevention science – in Europe and beyond.

COVID-19 has taught us how many lives can be lost due to misinformation, pseudoscience and inefficient narratives and false claims about science. This is why the Journal of Prevention will keep high standards on what we shall call “effective” or “promising” interventions but also give space to debates on upcoming topics, and a forum for those who work in applied prevention so that the practical relevance and the concrete usefulness of prevention sciences can be explained and communicated as well.

One of the first messages we received from our Ukrainian colleagues in the first days of the attack on Ukraine was an appeal to help them get their children out of the country into safety. The same happened when the Spanish democracy was attacked in 1936. It is an intrinsic and innate feature of human nature to first worry and think about the safety and wellbeing of children. And this is our mission and driver: the prevention sciences can provide the ideas for action, tools and necessary innovation for everybody to be able to offer a livable future to their children, with and without the help of technology. If the Journal of Prevention succeeds in communicating this clearly and understandably, we can make a positive and optimistic contribution to our societies’ resilience in this gloomy period.

Finally, the researchers, practitioners and policymakers gathered in EUSPR have a need for meeting each other to exchange experiences and challenge each other’s studies in forum and debate, following the pandemic. The Journal of Prevention is for this society an opportunity to document, sustain and further disseminate these debates, implying a robust upgrade of the academic discourse on prevention in and beyond Europe. We invite you to share and contribute.

The Board of Directors,

European Society for Prevention Research (EUSPR).

https://euspr.org/.

